# Non-invasive Brain Stimulation of the Posterior Parietal Cortex Alters Postural Adaptation

**DOI:** 10.3389/fnhum.2020.00248

**Published:** 2020-06-26

**Authors:** David R. Young, Pranav J. Parikh, Charles S. Layne

**Affiliations:** ^1^Center for Neuromotor and Biomechanics Research, Department of Health and Human Performance, University of Houston, Houston, TX, United States; ^2^Center for Neuro-Engineering and Cognitive Science, University of Houston, Houston, TX, United States

**Keywords:** adaptation, transcranial direct current stimulation, posterior parietal cortex, posture, after-effects, sensory integration, proprioception

## Abstract

Effective central sensory integration of visual, vestibular, and proprioceptive information is required to promote adaptability in response to changes in the environment during postural control. Patients with a lesion in the posterior parietal cortex (PPC) have an impaired ability to form an internal representation of body position, an important factor for postural control and adaptation. Suppression of PPC excitability has also been shown to decrease postural stability in some contexts. As of yet, it is unknown whether stimulation of the PPC may influence postural adaptation. This investigation aimed to identify whether transcranial direct current stimulation (tDCS) of the bilateral PPC could modulate postural adaptation in response to a bipedal incline postural adaptation task. Using young, healthy subjects, we delivered tDCS over bilateral PPC followed by bouts of inclined stance (incline-interventions). Analysis of postural after-effects identified differences between stimulation conditions for maximum lean after-effect (LAE; *p* = 0.005) as well as a significant interaction between condition and measurement period for the average position (*p* = 0.03). We identified impaired postural adaptability following both active stimulation conditions. Results reinforce the notion that the PPC is involved in motor adaptation and extend this line of research to the realm of standing posture. The results further highlight the role of the bilateral PPC in utilizing sensory feedback to update one’s internal representation of verticality and demonstrates the diffuse regions of the brain that are involved in postural control and adaptation. This information improves our understanding of the role of the cortex in postural control, highlighting the potential for the PPC as a target for sensorimotor rehabilitation.

## Introduction

Central integration of visual, vestibular, and proprioceptive sensory information is critical for successful postural control and the maintenance of upright stance (Peterka, 2002). Another important component of successful postural control is adaptability. Postural adaptation requires the updating of one’s internal representation of their position and movement within the environment (Head and Holmes, 1911; Chritchley, 1953). The internal representation can adapt in response to changes in sensory feedback and/or the external environment. These changes occur slowly and correspond with changes in behavior which gradually reduce movement errors (Gurfinkel et al., 1995). Once original conditions are restored, there is an after-effect while the internal representation recalibrates to its previous state (Kluzik et al., 2005; Ivanenko and Gurfinkel, 2018). After-effects dissipate over the course of seconds to minutes as prior experience and sensory feedback reverts the adapted internal representation to baseline (Wierzbicka et al., 1998; Kluzik et al., 2005). This investigation sought to improve our general understanding of how the posterior parietal cortex (PPC) is involved in postural adaptation.

Multisensory integration is impaired in individuals with lesions of the PPC (Derouesné et al., 1984). This suggests that the PPC is a sensory association area, where signals from multiple sensory systems (i.e., the visual, vestibular, and somatosensory systems) are integrated (Edwards et al., 2019). The PPC performs calculations, transforming sensory signals into sensorimotor representations of the body position to create an internal representation of our position in space (Sober and Sabes, 2003; Sabes, 2011; Findlater et al., 2016).

There is some evidence of the left hemisphere parietal lobe dominance in motor adaptation. Specifically, Mutha et al. (2011) identified that brain damage to the left PPC (lPPC) decreased visuomotor adaptation but damage to the right PPC (rPPC) did not. Newport et al. (2006) found that a bilateral lesion of the PPC, primarily in the left hemisphere, led to an inability to adapt to visual perturbations in a pair of 2006 case studies (Newport and Jackson, 2006; Newport et al., 2006). Other investigators have shown that disruptive TMS of the left PPC can impair adaptive reaching during a right-handed task (Desmurget et al., 1999). Alternatively, there is evidence that the right hemisphere parietal lobe, as part of a network with the right inferior frontal cortex, dominates processing of positional illusions induced by tendon vibration (Naito et al., 2007; Takeuchi et al., 2019). Still, others have found some level of bilateral activity associated with positional illusions generated by tendon vibration (Amemiya and Naito, 2016; Naito et al., 2016).

While the effects of brain stimulation of the PPC have yet to be explored regarding postural adaptation, previous research has demonstrated PPC involvement during postural control tasks with additional sensory integration demands (Ishigaki et al., 2016; Kaulmann et al., 2017). Both Ishigaki et al. (2016) and Kaulmann et al. (2017) identified that inhibition of the PPC *via* non-invasive brain stimulation altered the effects of augmented sensory feedback on postural stability. There is also evidence that the bilateral PPC is involved in continuous postural control during periods of sensory conflict (Goel et al., 2019). Based on the fact that the PPC is involved in upper body motor adaptation, processing of proprioceptive perturbations, and sensory integration during postural control, it is reasonable to hypothesize that the PPC is also involved in postural adaptation. Furthermore, a previous investigation by Heinen et al. (2016) identified that bilateral stimulation of the PPC was more effective at eliciting changes in working memory than unilateral stimulation. Thus, the bilateral role of the PPC should be investigated within the scope of postural adaptation. It is important to understand if there are hemisphere-specific roles of the PPC in postural adaptation or if the involvement is part of a more diffuse cortical network that requires bilateral PPC input.

To identify if relative facilitation or inhibition of the PPC alters postural adaptation, we employed bilateral transcranial direct current stimulation (tDCS). tDCS provides low-intensity stimulation, flowing from anodal to the cathodal electrode(s), which results in slight alterations in the excitability of underlying cortical tissue (Lefaucheur and Wendling, 2019). Anodal stimulation leads to a relative excitation of the underlying tissue while cathodal stimulation leads to a relative depression. Sham stimulation does not alter cortical excitability (Lefaucheur and Wendling, 2019). While there is no consensus, some previous investigations have found behavioral differences between sham and cathodal, but not a sham and anodal stimulation (Grundmann et al., 2011; Foerster et al., 2017). Improving the understanding of the PPC’s role in postural adaptation will improve the basic understanding of cortical influences on postural control and may have clinical implications for use of non-invasive brain stimulation to improve adaptability in fall-risk populations.

To identify differences in postural adaptation resulting from tDCS of the PPC, this investigation utilized an incline-intervention adaptation paradigm. Incline-interventions involve prolonged stance on an inclined surface and result in a postural after-effect known as lean after-effect (LAE), which is an anterior shift in position that can persist for several minutes (Kluzik et al., 2005; Chong et al., 2014, 2017). Lean after-effect reflects a change in the internal relationship between gravitational vertical and elected postural orientation. This incongruence is corrected over time as subjects reorient to gravity (Kluzik et al., 2005). The current investigation sought to determine the effects of bilateral tDCS stimulation of the PPC on adaptation to the inclined surface, as well as on de-adaptation once conditions return to normal. It was hypothesized that active tDCS would alter LAE, however, based on previous literature, it was not feasible to hypothesize about hemisphere-specific effects.

## Materials and Methods

### Subjects

Fifteen subjects were recruited to perform postural control tasks after tDCS stimulation across three data collection sessions. An additional 15 subjects participated in a control experiment without stimulation. All subjects provided their written informed consent following the Helsinki Declaration. Consenting documents were approved by the University of Houston institutional review board for experimental studies. Inclusion criteria included subjects being between 18–35 years of age, ability to stand without assistance, no history of neurological or musculoskeletal dysfunction, and no known contraindications to tDCS stimulation such as metallic implants, history of seizures or brain damage (Datta et al., 2011). Physical preparedness was assessed using a PAR-Q (Thomas et al., 1992).

### Protocol

**Figure 1:**
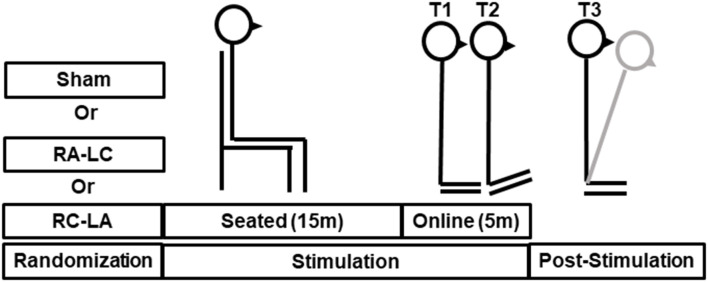
Illustrative representation of stimulation and tasks during T1 (baseline), T2 (incline-intervention) and T3, (lean after-effect period). Transcranial direct current stimulation (tDCS) stimulation was started offline while the subject remained quietly seated. After 15 min the subject immediately proceeded through the T1-T3 tasks. The total stimulation duration was 20 min. The gray line shown during T3 reflects a typical response to incline-intervention, lean after-effect (LAE).

**Figure 2:**
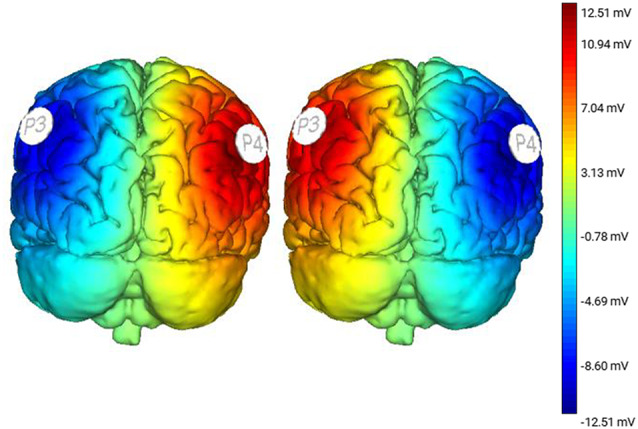
Stimulation modeling illustrating modeled changes in cortical excitability resulting from Right Anodal-Left Cathodal (RA-LC) and Right Cathodal-LeftAnodal (RC-LA) stimulation.

Subjects participated in three sessions, which were separated by a minimum of 48 h. The greatest period between sessions was 14 days [average 5.3 ± 4.1 days (mean ± SD)]. During each session, subjects performed an incline-intervention, which consisted of three trials (Figure 1). First, subjects performed a 30 s baseline trial of quiet stance (T1) on a horizontal surface. Next, they moved atop an inclined surface set to an angle of 10° for 5 min (T2). Last, subjects returned to standing on the horizontal surface (T3) where they stood for a final 5 min (Figure 1). Throughout the task, subjects were instructed to keep their eyes closed, place their arms across their chest, and stand naturally without, “resisting any pulls they felt on their body or temptation to lean.” tDCS was applied at the beginning of each session and was administered in random order. Stimulation was administered in three conditions: Right Anodal-Left Cathodal (RA-LC), designed to slightly depolarize tissue in the rPPC while slightly hyperpolarizing tissue in the lPPC. Right Cathodal-Left Anodal (RC-LA), designed to do the opposite, and Sham, where the current was ramped up for 30 s and ramped down after 30 s of stimulation to simulate the scalp sensation of active stimulation without injecting sufficient current to alter cortical excitability (Table 1). For all conditions, stimulation was initiated before the incline-intervention (i.e., before T1). For the first 15 min of stimulation, the subject sat quietly, then, at the 15-min mark, subjects began to perform the protocol. First, subjects performed the baseline trial (T1), then immediately moved atop the inclined surface for T2, then immediately began T3. Stimulation was terminated at the end of T2, resulting in a total stimulation duration of 20 m. The stimulation order was double-blinded to the subject and the administrator of the experiment. A stimulation model can be seen in Figure 2.

**Table 1:** Definitions.

Center of graviy	COG	Approximation of one’s overall position in space
Lean after-effect	LAE	Postural after-effect indicating adaptation, specifically adaptation to an incline-intervention
Posterior parietal cortex	PPC	Hub of multisensory integration in the cortex
Transcranial direct current stimulation	tDCS	Leads to depolarization or hyperpolarization of local brain tissue based on stimulation condition
Cathodal	RC or LC	Leads to relative hyperpolarization
Anodal	RA or LA	Leads to relative depolarization
Sham	Sham	Placebo

### Instrumentation

Incline-interventions were performed on a surface set to an incline angle of 10° (ASAHI Corporation, Gifu, Japan). Kinematic experimental data were collected using a 12-Camera Vicon motion capture system. Subjects were measured and outfitted with reflective markers based on Vicon Nexus’s Full Body Plug-in Gait Marker Set (Vicon, Oxford, UK). Kinematic data from all trials were captured at a rate of 100 Hz. Subjects also wore earmuffs to minimize auditory feedback. tDCS stimulation was performed using an eight-channel Starstim tDCS Device (Neuroelectrics, Spain). Saline soaked 25 cm^2^ sponges were placed at P3 and P4 using the international 10-20 system (Homan et al., 1987). For both active conditions, stimulation was applied at 1.5 mA.

### Data Processing

Kinematic data collected during the experiment were exported from Vicon Nexus and analyzed using custom MATLAB scripts (Mathworks, Inc., Natick, MA, USA). Marker trajectories of the legs and torso were utilized to compute the anterior-posterior (AP) center of gravity (COG) measurement. Based on previous literature, data derived from incline-interventions was filtered using a 4th order low-pass Butterworth filter with a cut-off frequency of 0.1 Hz. This design can isolate changes in the mean center of gravity while eliminating signal higher frequency COG fluctuations during prolonged trials (Kluzik et al., 2005).

T1 AP-COG was baseline corrected. Raw signals were set to a position of zero at the start of T1. Because the subjects physically moved to and from the inclined surface to undergo T2, T3 AP-COG measures also required a baseline correction. To achieve this, upon returning to a horizontal stance, subjects stood with their eyes open for 5 s. Data during this time was averaged and that average was set to zero to ascertain lean after-effect related shift in COG while ignoring minute changes that may have occurred while the subject moved to and from the inclined surface. The subjects’ eyes remained open during the initial 5 s of T3 because the opening of the eyes has been shown to extinguish LAE (Earhart et al., 2010). Data reflected that this was the case, with lean after-effect onset occurring once the eyes were closed. Thus, any anterior measure of COG is relative to the pre-adaptation stance, not an absolute position. We conducted a control experiment where 15 additional participants experienced the incline-intervention (i.e., T1-T3) without receiving tDCS to determine if experiencing active or sham tDCS influenced baseline (i.e., flat surface) stance.

**Figure 3:**
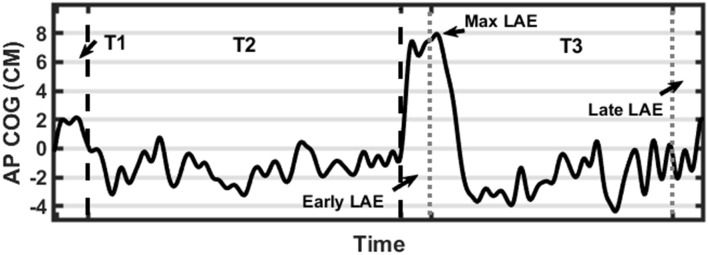
Graphical representation of data processing parameters. Black dashed lines indicate transitions between trials (first line from T1-T2 and a second line from T2-T3). Gray dotted lines indicate measurement periods reflecting average anterior-posterior center of gravity (AP-COG) in during Early and Late LAE periods. The data presented are from one representative trial.

We computed the mean position (Average AP-COG), the standard deviation of position, path length, and root mean square of position in the AP direction to compare baseline stance between stimulation conditions and the control condition (unstimulated). The COG data derived from the baseline-corrected post-inclined stance (T3) was used to calculate several outcome measures reflecting the magnitude of postural adaptation (LAE). Before any further calculations, two time periods were identified, the first 30 s of T3 was defined as the Early LAE period while the final 30 s was defined as the Late LAE period. The Max LAE was also calculated, which was defined as the maximum anterior AP-COG. Average AP-COG (Ave-COG) position during the Early and Late periods of T3 was also calculated to identify the magnitude of LAE present during each period. Figure 2 describes these outcome measures visually (Figure 3). Finally, Off-Set Time, the first sample following Max LAE in which the subject returned to an average position within two standard deviations (SD) of their baseline position for a period of 10 s was calculated to identify what, if any, effect stimulation condition had on the time-course of recalibration to upright stance (Kluzik et al., 2005, 2007).

### Statistical Analysis

To verify that tDCS did not alter baseline stance, average position in the AP direction, the standard deviation of AP position, AP-path length, and root mean square (RMS) of AP position were compared between stimulation conditions during T1. An additional sample of baseline measures from 15 subjects who did not receive tDCS stimulation was also included in the comparison to verify that Sham stimulation did not alter an unperturbed stance. Comparisons were made using separate repeated measures analysis of variance (rm-ANOVA) for each variable. Next, to identify the effects of tDCS stimulation on lean after-effect (LAE), a two-way rm-ANOVA (Condition by Time) was performed to compare average AP-COG during three time periods. First, the baseline position (T1), next during the Early period of T3, and last during the Late period of T3 to compare lean after-effect between the stimulation conditions. Follow-up one-way ANOVAs were utilized to compare average AP-COG during Early and Late LAE periods as well as for Max LAE and Off-Set Time between stimulation conditions. Pairwise comparisons for analyses were made using Bonferroni *post hoc* adjustments. For all analyses, significant findings were defined by an alpha value of *p* < 0.05. Effect sizes, derived from partial eta squared ($\eta_{\text{p}}^{2}$) for main effects and Hedge’s G (HG) for pairwise differences were also derived in cases of significant findings. Statistical analyses were performed using SPSS (Version 25.0. IBM Corp., Armonk, NY, USA).

## Results

Fifteen subjects, eight females and seven males completed the study. Subjects were aged 23.4 ± 4.2 years, were 165.6 ± 12.6 cm tall, and weighed 77.4 ± 18.3 kg. When asked at the end of each session to identify what stimulation condition they had received, subjects guessed correctly 1.0 ± 0.78 times out of three indicating that subjects were generally unaware of what stimulation condition they were experiencing. Results of one-way rm-ANOVAs revealed no difference between stimulation conditions for the mean position (*F*_(3,12)_ = 0.59, *p* = 0.63, *η*^2^ =0.13), standard deviation of position (*F*_(3,12)_ = 0.24, *p* = 0.74, *η*^2^ = 0.09), AP path length (*F*_(3,12)_ = 0.14, *p* = 0.93, *η*^2^ = 0.03) or RMS of AP position (*F*_(3,12)_ = 0.29, *p* = 0.83, *η*^2^ = 0.07) during baseline (T1) trials. This analysis included results from 15 pilot subjects who received no brain stimulation (i.e., unstimulated), demonstrating that tDCS did not alter the characteristics of stance during the baseline trial T1 and that the Sham condition can serve as effective control.

**Figure 4:**
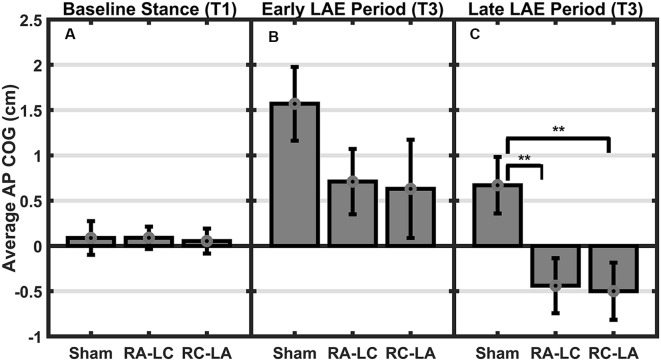
Mean AP COG ± 1 SEM. Active stimulation significantly altered the lean after-effect. A significant effect of condition (*p* = 0.008) and condition by time interaction effect (*p* = 0.03) was found. No differences were observed during baseline stance (*p* = 0.63; **A**). A significant main effect of condition on average LAE during the early period **(B)** was found (*p* = 0.046) but no pairwise differences. Both active conditions exhibited lesser LAE than Sham during the Late LAE **(C)** period than Sham (*p* = 0.004; RA-LC *p* = 0.008 RC-LA *p* = 0.003), **p < 0.01.

**Figure 5:**
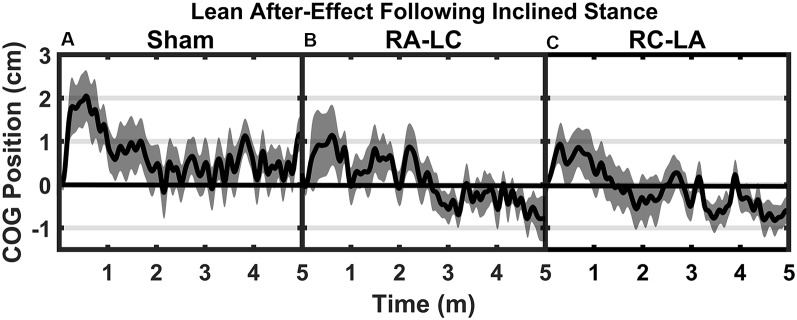
Mean AP COG ± 1 SEM position during T3 after Sham **(A)**, RA-LC **(B)**, and RC-LA **(C)** stimulation.

**Figure 6:**
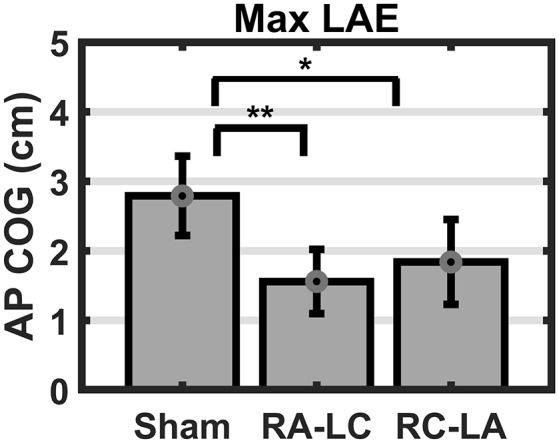
Mean AP COG ±1 SEM. Active stimulation decreased the maximum lean after-effect (*p* = 0.005). Both active conditions exhibited smaller Max LAE than Sham (RA-LC *p* = 0.009; RC-LA *p* = 0.03), *p < 0.05, **p < 0.01.

Analysis of COG data derived from the lean after-effect periods revealed that tDCS stimulation altered responses to inclined stance. Individual data can be found in Table 2. This is shown by differences in lean after-effect during T3 (Figure 4). Results of a two-way rm-ANOVA comparing Ave-COG during Baseline, Early and Late LAE periods between stimulation conditions revealed a significant overall effect of condition (*F*_(2,13)_ = 7.61, *p* = 0.008, *η*^2^ = 0.52) no significant effect of time (*F*_(1,14)_ = 5.2, *p* = 0.19, *η*^2^ = 0.23) but a significant interaction effect between time and condition (*F*_(2,13)_ = 3.96, *p* = 0.03, *η*^2^ = 0.59). Additional analyses identified a significant main effect of stimulation conditions for Ave-COG during Early LAE (*F*_(2,13)_ = 3.93, *p* = 0.046, *η*^2^ = 0.38), but pairwise comparisons using Bonferroni *post hoc* adjustments revealed no specific differences between stimulation conditions (Sham to RA-LC *p* = 0.07, Sham to RC-LA *p* = 0.14, RA-LC to RC-LA *p* = 1; Figure 4B). The condition also influenced the Ave-COG during the Late LAE period (*F*_(2,13)_ = 8.47, *p* = 0.004, *η*^2^ = 0.57). Subsequent pairwise comparisons revealed that the RA-LC and RC-LA conditions each exhibited significantly less Ave-COG during Late LAE compared to the Sham condition (*p* = 0.008, HG = 0.94; *p* = 0.003, HG = 0.98, respectively). The Ave-COG in the Late LAE for both active stimulation conditions were posterior to baseline (Figure 4C). Again, the average COG during the Late LAE period was not different between active conditions (*p* = 1). Time series data representing the lean after-effect between conditions can be observed in (Figure 5). A one-way rm-ANOVA of the maximum AP-COG (Max COG) again revealed a main effect of tDCS stimulation (*F*_(2,13)_ = 8.33, *p* = 0.005, *η*^2^ = 0.356; Figure 6). *Post hoc* comparisons found that both active stimulation conditions exhibited significantly less maximum forward lean than Sham (RA-LC *p* = 0.009 HG = 0.61; RC-LA *p* = 0.03 HG = 0.42), but there was again no difference between the two-active stimulation conditions (RA-LC to RC-LA *p* = 1). There were no differences in Off-Set Time between the three stimulation conditions (*F*_(2,13)_ = 0.99, *p* = 0.397, *η*^2^ = 0.13).

**Table 2:** Postural adaptation outcomes.

	Maximum LAE			Average COG early			Average COG late			Off-set time		
Participant	Sham	RA-LC	RC-LA	Sham	RA-LC	RC-LA	Sham	RA-LC	RC-LA	Sham	RA-LC	RC-LA
1	2.44	0.95	0.63	1.00	0.09	0.57	0.31	0.02	-0.45	55.98	57.17	8.55
2	1.09	1.80	2.71	-1.40	-1.51	-0.55	1.71	-1.47	-1.43	37.28	50.92	48.55
3	1.17	1.88	1.45	0.80	1.60	0.39	0.82	0.30	0.53	42.34	42.25	0.55
4	2.34	1.15	1.19	0.44	0.27	-0.13	-1.21	-0.73	-2.63	13.83	11.61	13.41
5	5.01	1.43	2.90	4.79	1.14	-0.47	1.76	-0.74	0.20	37.42	5.87	38.43
6	3.60	0.82	0.47	2.15	0.13	-3.79	2.44	-1.57	-0.76	300	0.34	0.02
7	3.71	3.01	1.88	2.43	1.41	0.99	1.05	-1.52	0.43	36.55	63.48	166.99
8	8.73	5.52	9.41	6.05	2.89	8.50	2.01	-2.47	-4.93	61.71	72.38	115.13
9	1.17	3.02	2.52	0.59	0.63	2.30	0.04	-0.68	-0.18	117.91	172.19	92.77
10	2.93	1.98	2.14	1.47	0.62	0.53	1.12	-0.54	-0.64	230.14	257	76.61
11	0.27	-0.24	-0.34	0.13	-0.33	-0.99	-0.99	-1.47	-2.03	0.69	0.02	0.02
12	5.41	4.75	4.52	1.16	0.74	1.36	3.51	1.17	3.00	300	173.64	180.15
13	1.24	0.82	1.00	0.72	0.50	0.26	0.72	0.34	-0.27	122.14	48.37	39.49
14	2.72	2.69	1.06	0.65	1.41	-0.09	2.94	1.04	-1.75	55.81	38.76	77.09
15	2.15	0.15	0.50	1.02	-0.30	-1.00	0.50	0.22	1.33	45.57	17.35	22.95
Mean	2.93	1.98	2.14	1.47	0.62	0.53	1.12	-0.54	-0.64	97.16	67.42	58.71
SD	2.17	1.60	2.35	1.84	1.02	2.59	1.33	1.05	1.83	99.44	75.07	58.84

## Discussion

The current investigation was designed to assess the role of the PPC in postural adaptation within a group of 15 healthy, young adults. Neuromodulation was applied in three conditions: RA-LC, RC-LA, and Sham in a random, double-blind fashion. Postural adaptation, measured by lean after-effect, was decreased in both active stimulation conditions compared to Sham. These findings demonstrate that active stimulation decreased initial adaptation to the incline-intervention (i.e., less forward lean). Off-Set Time was not different between conditions, which shows that while the magnitude of the adaptation was affected, the time course necessary to re-orient to gravity was not. These findings suggest impaired adaptability of the internal representation of one’s body position following active stimulation of the PPC, regardless of which hemisphere was inhibited and which was excited. Additionally, the lack of differences between conditions in the Off-Set Time measure suggests that the time course associated with return to baseline may result from central processing occurring in areas of the cortex other than the PPC.

Evidence indicates that PPC integrates sensory information from the visual, vestibular, and proprioceptive systems to maintain an internal representation of one’s posture, which is continuously updated to influence motor commands (Andersen et al., 2004; Buneo and Andersen, 2006). During this experiment, we observed no difference in LAE between active stimulation conditions (i.e., RA-LC and RC-LA). This finding is of interest because some experiments have sought to identify hemisphere-specific roles of the PPC. Studies have suggested that the right hemisphere may be activated to a greater extent than the left in response to proprioceptive manipulations leading to movement illusions, such as tendon vibration (Hagura et al., 2009; Amemiya and Naito, 2016; Naito et al., 2016, 2017). In a series of studies, Naito et al. (2017) identified increased activity in the right inferior parietal lobule (IPL) during tendon vibration, which they identify as part of a frontoparietal network involved in proprioceptive processing to maintain a sense of position. According to the same group, the left IPL may be biased towards computations that associate self-position and the external environment and may be less sensitive to proprioceptive perturbations (Naito et al., 2016). Conversely, the left hemisphere PPC has been suggested to be an area closely associated with motor adaptation.

In a pair of case studies, Newport et al. (2006) identified impaired prism adaptation in a patient with bilateral damage to the PPC, with greater damage in the left hemisphere (Newport and Jackson, 2006; Newport et al., 2006). Later, these results were clarified by Mutha et al. (2011). In their study, Mutha et al. (2011) found that visuomotor adaptation was hindered in subjects with lesions of the left, but not right parietal region. The authors argued that the left parietal region is involved in modifying the internal representation of self-position, and the relationship between movement and the environment (Mutha et al., 2011). While Mutha et al. (2011) suggested that the left parietal region was more important for visuomotor adaptation, the current investigation found decreased postural adaptation through inhibition of either hemisphere. There are several possible reasons for these findings.

For many visuomotor adaptation experiments, a unilateral upper limb task has been employed, which could induce greater activation in the contralateral hemisphere than a bilateral task. Simply by the nature of postural control as a bilateral task, there may be increased bilateral input from several cortical areas. Postural adaptation tasks also include stability demands, unlike upper body adaptation tasks. Sensory processing may be altered by additional stability related requirements including the updating of the internal representation of the body and its relationship to gravitational space. Previous investigations have found increased PPC activation during difficult postural control tasks when exposed to multiple sensory perturbations (Takakura et al., 2015). Unfortunately, Takakura et al. (2015) were only able to record hemodynamics from the right hemisphere and were unable to identify if bilateral increases in activity were present. In healthy subjects, Ishigaki et al. (2016) found altered sensory integration of augmented feedback from cathodal tDCS stimulation of the lPPC during postural control, however, their study utilized light touch only on the right hand (Ishigaki et al., 2016). Johannsen et al. (2015) also identified that normal postural control involves parietal representation and that modulation of these regions can alter postural dynamics during periods of altered sensory feedback.

As the PPC has not previously been studied through the lens of postural adaptation, it was not apparent if there would be hemisphere specific contributions in response to a postural incline task. While the results of the current investigation cannot delineate the specific roles of the hemispheres, the results suggest that both hemispheres are involved in postural adaptation. This may be due to the reciprocal connections which exist between the bilateral PPC and the cerebellum (Amino et al., 2001). Specifically, the IPL, which compares perceived body positions to extra personal space is innervated by the cerebellum (Clower et al., 2001). The PPC utilizes sensory feedback as well as the efferent copy provided by the cerebellum to maintain an internal representation of limb positions and the body in space, what could be described as body ownership or the body schema (Amino et al., 2001; Dijkerman and de Haan, 2007; Kammers et al., 2009; Parkinson et al., 2010; van Stralen et al., 2011). Recent publications have even gone so far as to identify the PPC as the home for the “posture cells of the brain” (Chen, 2018; Mimica et al., 2018). The current study reinforces the notion of the bilateral PPC’s role in monitoring and updating the internal representation.

This investigation identified decreased postural adaptation following active stimulation of the bilateral PPC. This study employed a paradigm that stimulated the bilateral PPC instead of placing the return electrode on another brain region (i.e., the supraorbital foramen; Ishigaki et al., 2016) to contain stimulation to the PPC and not alter the excitability of other brain regions such as the somatosensory or motor cortices. Because of this, as one hemisphere received inhibitory stimulation, the other received excitatory stimulation. Previously, some studies have shown that healthy young subjects experience alteration of sensory detection thresholds following cathodal, but not anodal stimulation of S1 (Grundmann et al., 2011). Additionally, it has been shown that cathodal stimulation of the cerebellum decreases postural stability while anodal does not change stability in young subjects (Foerster et al., 2017). Other studies have shown the effects of anodal, but not cathodal stimulation in healthy subjects when stimulation was performed over motor areas (Carter et al., 2015). Additionally, it is possible that by utilizing a stimulation paradigm that required current to cross between hemispheres, our stimulation led intra-hemispheric interactions that cannot be fully explained by describing the sum of anodal and cathodal stimulation (Lindenberg et al., 2013). There is also some evidence that the inhibitory stimulus can lead to improved performance. Inhibitory stimulation could suppress neural noise, which can improve the signal to noise ratio, and facilitate the function of the affected cortical structures (Antal et al., 2004; Filmer et al., 2013). Therefore, we can only speculate as to whether the decreased adaptability observed in our study is due to the inhibitory nature of cathodal stimulation, or excitatory nature of anodal stimulation. Regardless, the current study does demonstrate that stimulation designed to alter PPC excitability does alter adaptability overall. The present results also indicate that stimulation does not serve to disrupt the time course of postural de-adaptation suggesting the magnitude of LAE is a separate process from the temporal characteristics of LAE. Future investigations should be conducted to explore this possibility.

In the future, designs may confirm these findings using techniques such as disruptive transcranial magnetic stimulation (rTMS) because more focal stimulation is less likely to alter the excitability of other brain regions. A previous publication by Johannsen et al. (2015) has illustrated the possibility of the use of rTMS to investigate parietal processes involved in postural control. The group performed 1,200 pulses of inhibitory rTMS at a frequency of 1 Hz to disrupt the left inferior parietal gyrus and identified that this disruption altered the body-sway response to altered sensory feedback during the posture. These results verify the possibility of using rTMS to probe the parietal lobes in the field of postural control (Johannsen et al., 2015). Also, while this study involved neuromodulation of the PPC, no recordings of brain activity were obtained. Future investigations are needed to verify the effects of tDCS on PPC excitability in postural adaptation. The brain is a complex and dynamic organ. As such, the brain’s state during stimulation can influence the effects of non-invasive brain stimulation. Future research should endeavor to utilize neuroimaging (i.e., EEG) to optimize stimulation parameters to elicit the desired cortical changes (Bergmann, 2018). This study is important because while upper body motor adaptation research is critical for understanding the dynamics of human sensorimotor control, postural adaptation is more directly linked to public health due to fall risk. Therefore, this investigation provides novel information that may lead future experimentation to what efficacy there may be for non-invasive brain stimulation as a therapeutic measure to improve adaptability during postural control, decreasing fall risk. Future investigations should include clinical populations to identify the viability of tDCS of the PPC as a rehabilitative mechanism as well as include neuroimaging techniques.

## Data Availability Statement

The original contributions presented in the study are included in the article (Table 2), further inquiries can be directed to the corresponding author.

## Ethics Statement

The studies involving human participants were reviewed and approved by University of Houston Committee for the Protection of Human Subjects. The patients/participants provided their written informed consent to participate in this study.

## Author Contributions

DY, PP, and CL contributed to the study design. DY recruited the subjects and was involved in the informed consent process, processed, and analyzed data and wrote the first draft of the manuscript. All authors contributed to data interpretation and manuscript revision, and all read and approved the submitted version.

## Conflict of Interest

The authors declare that the research was conducted in the absence of any commercial or financial relationships that could be construed as a potential conflict of interest.
